# Automatic Recognition of Dual-Component Radar Signals Based on Deep Learning

**DOI:** 10.3390/s25061809

**Published:** 2025-03-14

**Authors:** Zeyu Tang, Hong Shen, Chan-Tong Lam

**Affiliations:** 1 Faculty of Applied Sciences, Macao Polytechnic University, Macao SAR 999078, Chinactlam@mpu.edu.mo (C.-T.L.); 2 School of Engineering and Technology, Central Queensland University, Brisbane 4000, Australia

**Keywords:** pulse-internal modulation classification, dual-component pulse-internal modulation, multi-label learning, convolutional neural networks, multi-head self-attention

## Abstract

The increasing density and complexity of electromagnetic signals have brought new challenges to multi-component radar signal recognition. To address the problem of low recognition accuracy under low signal-to-noise ratios (SNR) in adapting the common recognition framework of combining time–frequency transformations (TFTs) with convolutional neural networks (CNNs), this paper proposes a new dual-component radar signal recognition framework (TFGM-RMNet) that combines a deep time–frequency generation module with a Transformer-based residual network. First, the received noisy signal is preprocessed. Then, the deep time–frequency generation module is used to learn the complete basis function to obtain various TF features of the time signal, and the corresponding time–frequency representation (TFR) is output under the supervision of high-quality images. Next, a ResNet combined with cascaded multi-head attention (MHSA) is applied to extract local and global features from the TFR. Finally, modulation format prediction is achieved through multi-label classification. The proposed framework does not require explicit TFT during testing, and the TFT process is built into TFGM to replace the traditional TFT. The classification results and ideal TFR are obtained during testing, realizing an end-to-end deep learning (DL) framework. The simulation results show that, when SNR > −8 dB, this method can achieve an average recognition accuracy close to 100%. It achieves 97% accuracy even at an SNR of −10 dB. At the same time, under low SNR, the recognition performance is better than the existing algorithms including DCNN-RAMIML, DCNN-MLL, and DCNN-MIML.

## 1. Introduction

Signal modulation recognition is a key technology of great significance in both civilian and military fields. In the civilian field, it is widely used in the monitoring and management of the electromagnetic spectrum to ensure communication security and the effective control of signals. In the military context, it is used to assess the threat level of enemy equipment and assist in the operation of military reconnaissance equipment by identifying the types and parameters of electromagnetic signals [1,2]. However, with the rapid development of information technology, the current electromagnetic environment is increasingly complex [3], which brings huge challenges to radar signal modulation identification technology, especially in the case of dense signals, multi-component signals may be generated. Therefore, effective analysis and identification of multi-component modulated radar signals under different signal-to-noise ratios (SNR) conditions is a more practical and challenging task.

Traditional radar signal recognition methods are divided into two types: those based on decision theory and those based on feature extraction. Methods based on decision theory [4,5] transform the signal identification problem into a hypothesis testing problem, relying on probability theory and Bayesian estimation theory. However, this method has strict requirements on the prior information of the signal and the algorithm is computationally intensive, which is not conducive to blind identification. Existing algorithms based on feature extraction have low computational complexity, by converting signal preprocessing into a certain transformation domain, and using classifiers for training and learning, such as artificial neural networks [6] and support vector machines [7]. However, the performance of algorithms based on feature extraction depends on the quality of the extracted features, and the selection of features often relies on the experience of the researcher and can only target relatively ideal conditions [8].

Signal modulation recognition based on deep learning (DL) can adaptively extract optimal features and greatly improve the recognition performance [9,10,11,12]. Many researchers have studied long short-term memory (LSTM), one-dimensional convolution, and deep neural network (DNN) algorithms for the feature extraction and classification of single-component time-domain radar signal modulation recognition tasks, and achieved good performance under low SNR conditions [13,14]. With the rapid development of CNN in the field of image processing, research on converting one-dimensional time-domain radar signals into two-dimensional time-frequency images (TFIs), and then using CNN networks to extract and classify radar signal time-frequency images has also received increasing attention. Jiang et al. [15] used multi-layer decomposition to denoise the signal and obtained the signal time–frequency image through Choi–Williams distribution (CWD). Finally, they used an improved convolutional neural network to classify 12 types of signals. In another study [16], the author composed three complex modules based on CNN into LPI-Net, which was used to learn the texture features of CWD time–frequency images. In addition, Jiang et al. [17] used smoothed pseudo WVD (SPWVD) to convert radar signals into TFIs. Then, the local dense connection U-net is introduced as a denoising network to denoise the time–frequency image to assist the deep convolutional neural network (DCNN) network for recognition. In order to obtain high-quality TFIs, image processing techniques such as image filtering and cutting were used in the literature [18] to remove background noise and redundant frequency bands, and obtain grayscale images containing the main morphological features. The preprocessed image is then input into the ACDCA-ResNeXt network to achieve recognition.

However, as can be seen from the above, its limitation is that the classification of intra-pulse modulation mainly focuses on single-component intra-pulse modulation, and the conditions are relatively ideal. With the increasing complexity of intra-pulse modulation technology, during the process of electronic countermeasures, the receiver may receive multiple signals at the same time, causing the received signals to overlap in the time domain and frequency domain. Therefore, it is necessary to consider the occurrence of multi-component intra-pulse modulation in the electromagnetic space. In recent years, some researchers have proposed methods such as blind source separation [19] and parameterized time–frequency analysis (TFA) [20], which have achieved good performance. In recent years, with the rapid development of deep learning technology, some researchers have proposed a multi-component radar signal modulation recognition method based on deep learning. Among them, the research on multi-component radar signal recognition using the time–frequency images of radar signals combined with CNN has become a hot topic. Pan et al. [21] designed a multi-instance multi-label learning framework based on deep CNN, combining SPWVD time–frequency diagram with MIML-DCNN to realize the recognition of simulated overlapping signals of four different modulation types. SI et al. [22] proposed a new multi-class learning framework based on SPWVD and DCNN, which enhanced the connection between network modules by introducing the MBconv module and reduced the transmission loss of features. In order to better highlight the time–frequency characteristics of radar signals, ref. [23] uses the improved Cohen-type time–frequency distribution (CTFD) to generate time–frequency images. Then, three semantic segmentation networks, fully convolutional neural network (FCN-8s), U-Net, and DeepLab V3, were used to separate and identify the signals. Under low SNR, time–frequency images are susceptible to noise contamination. In ref. [24], the ResSwinT network is employed to denoise and reconstruct dual-component time–frequency images at various SNRs, and the SwinT network is used to recognize dual-component radar signals with random combinations among 12 modulation formats.

In summary, most of the existing multi-component modulation recognition works target TFIs for recognition, using CNNs as the backbone feature extraction network, commonly adopting the TFI-CNN model. Therefore, TFA has become an indispensable part of multi-component radar signal research. TFA has been developed over several decades, producing many classic algorithms. Ebrahim Ghaderpour [25] demonstrated the potential of the least squares wavelet (LSWAVE) software (https://www.mathworks.com/matlabcentral/fileexchange/70526-lswave-signalprocessing, accessed on 9 March 2025) [26] for analyzing VLBI time series and coherence analysis based on least squares wavelet analysis (LSWA). Mateusz et al. [27] studied the performance of empirical mode decomposition (EMD) and singular spectrum analysis (SSA) in the detection of aerodynamic instability in centrifugal compressors. Liu et al. [28] used spectrum reconstruction technology to study a denoising method for random noise in active source seismic data. TFA such as short-time Fourier transform (STFT), Wigner–Ville distribution (WVD), and SPWVD are also widely used in various fields. Some existing multi-component radar modulation recognition works directly use these TFA algorithms to obtain TFIs and then focus mainly on the preprocessing of TFIs and subsequent tasks. However, the process of converting time-domain multi-component signals into TFIs is often overlooked. For instance, refs. [21,22] applied SPWVD transform in recognition systems, while ref. [24] uses a denoising network to denoise and reconstruct TFIs. Although these methods can improve the recognition performance of multi-component radar signals, they also have the following issues:

1. Existing TFI-based multi-component radar signal recognition works all use the TFI-CNN recognition framework. During the testing phase, the entire recognition process includes multiple independent steps such as time–frequency transformation (TFT), denoising, feature extraction, and classification, which increases the risk of introducing errors, and no end-to-end system has been formed to implement this process.
2. It is difficult to obtain clear TFIs under low SNR conditions. The trend of the time–frequency ridges in the TFI reflects the changes in the signal’s instantaneous frequency over time, while different forms of time–frequency ridges display the intra-pulse characteristics of different signals. Traditional TFA is susceptible to noise interference, and under low SNR conditions, the time–frequency ridges of the signal in the TFI can easily become distorted. This blurs the intra-pulse characteristics and texture features of the signal in the time–frequency domain, thereby affecting recognition.
3. Recent works [22,23,24] have utilized advanced networks to denoise and reconstruct features of the TFIs, thereby obtaining clear TFIs. These denoising networks typically operate on the transformed noisy time–frequency images. Although this method improves accuracy compared to the traditional TFI-CNN method, the subsequent recognition network does not fully utilize the original time-domain information throughout the recognition process, but only works on the denoised TFI. Additionally, under low SNR conditions, some signals often fail to exhibit complete intra-pulse characteristics in the time–frequency domain due to noise interference. This can lead to confusion between signal intra-pulse characteristics and noise, causing the denoising network to misinterpret the signal.
4. Most current work uses deep CNNs to classify radar signal TFI. Convolutional networks are both local and translation invariant, allowing convolution operations to learn more edges and higher-level local features of objects in images. However, the locality of convolution operations also limits their ability to learn global features in images and signal location information features, which are critical for obtaining satisfactory recognition results at low SNR.

Therefore, we propose the TFGM-RMNet dual-component radar signal recognition framework. Unlike the previous TFI-CNN multi-component recognition scheme, TFGM-RMNet automatically learns to generate TFRs through the TFGM module for multi-component radar signals, effectively alleviating the low-quality TFI generated by traditional TFA under low SNR conditions. Specifically, this framework mainly consists of a deep time-frequency generation module and a classification module. The noise signal is preprocessed first and then used as the input sample for TFGM, with the noise-free TFI as the learning target. During this process, the TFGM module guides the network weights to adaptively learn basis functions to obtain various TF features and reconstruct them to generate the TFR. Due to the supervision from clean TFI, this also endows the TFGM module with the ability to perform automatic denoising. Finally, RMNet, which combines ResNet and Transformer learning, fully extracts local and global features from the TFR, and ultimately outputs category predictions. The main contributions of this paper can be summarized as follows:

1. For dual-component modulation recognition, a TFGM-RMNet network is proposed, which embeds the deep learning-based TFA module TFGM into the recognition network to replace the traditional TFA. In the testing phase, the end-to-end TFGM-RMNet directly generates the recognition results, avoiding the step-by-step operation in the traditional method and eliminating the need to design a denoising network, thereby achieving a performance improvement compared to the traditional multi-class radar signal recognition scheme.
2. TFGM consists of reduction, encoder, and decoder, which are responsible for frequency domain feature mapping, feature extraction, and reconstruction, respectively. The reduction module extracts the frequency domain features of the time domain signal by adaptively learning the basis function through convolution weights. The encoder and decoder aggregate the time–frequency features to generate TFR. In order to improve the quality of the generated TFR, we use mean square error (MSE) loss and perceptual loss for training to improve the pixel and structural similarity of TFR.
3. The classification network of our model adopts a hybrid design combining local convolution and global self-attention. Unlike the vision transformer (ViT), which divides images into blocks as input, our model replaces convolution with a cascaded multi-head self-attention (MHSA) layer to achieve global self-attention on the convolved 2D feature map, alleviating the problem of a lack of biases in the hidden layers of CNN that affect the recognition accuracy.
4. We conduct extensive experiments to verify the effectiveness of the proposed framework and compare the recognition accuracy of our model with existing work. Experimental results show that our model has good recognition performance under low SNR.

The remainder of this paper is organized as follows. Section 2 introduces the mathematical model of multi-component radar signals and the reassignment SPWVD (RSPWVD) algorithm. Section 3 describes the proposed TFGM-RMNet framework and explains each module in detail. Subsequently, in Section 4, we present the experimental dataset, implementation, and results. In Section 5, we provide a discussion. Finally, in Section 6, we conclude this paper.

## 2. Preliminaries

### 2.1. Signal Model

Assume there are *K* independent radar transmitters in the electronic warfare scenario, simultaneously emitting different types of LPI signals with various modulation schemes and closely spaced carrier frequencies. All transmitted radar signals are intercepted by the receiver, hence the received signals are likely to overlap in both the time and frequency domains. To simulate the real electromagnetic environment, this paper assumes that the overlapped signal y is affected by additive white Gaussian noise (AWGN). The SNR is defined as:

(1) $$\mathrm{SNR}=10\log_{10}\left(P_{s}/P_{w}\right)$$

where $P_{s}$ represents the signal power, and $P_{w}$ represents the AWGN power. The multi-component radar signal y(t) can be expressed as:

(2) $$y\left(t\right)=\sum_{i=1}^{k}s_{i}\left(t\right)+n\left(t\right)=\sum_{i=1}^{k}A_{i}\mathrm{rect}\left(t/T_{i}\right)e^{j\left(2\pi f_{c_{i}}t+\phi_{i}\left(t\right)+\phi_{0_{i}}\right)}+n\left(t\right)$$

where $S_{i}\left(t\right)$ represents the LPI radar signal emitted by the *i*th transmitter with a specific modulation type. n(t) denotes the AWGN. $A_{i}$ represents the non-zero amplitude of the *i*th component signal. Additionally, $T_{i}$ denotes the pulse width; $f_{c_{i}}$ stands for the carrier frequency; $\phi_{0_{i}}$ represents the initial phase; and $\phi_{i}\left(t\right)$ is the phase function.

Dual-component radar signals formed by the superposition of two types of radar modulation signals are widely present in practical electronic warfare scenarios. Therefore, this paper mainly considers the superposition of dual-component signals under the same SNR. In the signal model mentioned above, we set *k* to 2. Suppose there are eight types of single-component modulation signals, and overlapping signals are formed by randomly combining two types of single-component modulation signals. Then, the sample dataset can be represented as $D=\left\{\left(x_{i},x_{t}\right)|1\leq i\leq N\right\}\;$, where *N* represents the number of samples, and the *i*th sample is denoted as $x_{i}$. $t_{i}=\left[t_{i1},t_{i2},\ldots ,t_{i8}\right]$ represents the true label vector of $x_{i}$. When the *j*th modulation type appears in $x_{i}$, $t_{ij}=1$; otherwise, $t_{ij}=0$. For example, a label vector t=[0,1,0,0,0,1,0,0] for sample *x* means the second and sixth radar signals are combined in *x*. This paper considers 8 modulation methods, with a total of 36 combinations of dual-component signals.

### 2.2. Time–Frequency Transformation of Signals

**Figure 1:**
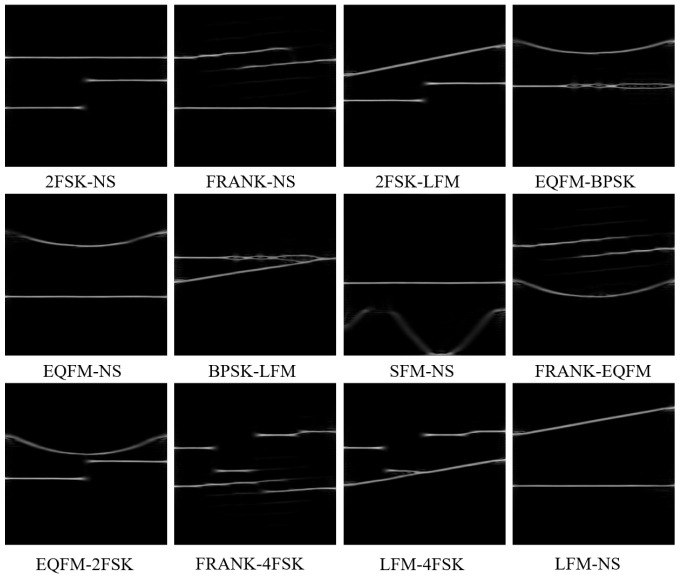
RSPWVD of 12 dual-component radar modulated signals without noise.

Time–frequency transformation is an important tool for analyzing non-stationary signals as they can compute the frequency components of a signal and represent the original time-domain signal as a two-dimensional function of frequency and time [29]. In the process of multi-component modulation recognition, TFT is commonly used in the preprocessing stage, where good time–frequency images can better represent signal characteristics, thus facilitating subsequent network recognition. However, due to the increasing complexity of electromagnetic environments, time-frequency representation (TFR) is often affected by noise. Therefore, researchers have tried various TFT methods for radar signal processing. The common TFT methods used in multi-component radar signal recognition include Choi–Williams distribution (CWD) [15,16], Wigner–Ville distribution (WVD), SPWVD [17,18], etc. Compared to WVD, SPWVD effectively prevents and eliminates cross-interference by improving smoothing algorithms in both the time and frequency domains. The SPWVD of a signal x(t) is defined by the equation:

(3) $$\mathrm{SPWV}D_{x}\left(t,\omega \right)=\int_{-\infty }^{+\infty }\int_{-\infty }^{+\infty }g\left(u\right)h\left(\tau \right)x\left(t-u+\frac{\tau }{2}\right)\times x^{*}\left(t-u-\frac{\tau }{2}\right)e^{-j\omega \tau }dud\tau$$

where g(u) and h(τ) are window functions used to reduce the time–frequency resolution, effectively reducing cross-terms. However, with increasing smoothing, there is a significant decrease in the time–frequency resolution. The RSPWVD algorithm introduces rearrangement technology based on the SPWVD algorithm to further suppress cross-terms while maintaining time–frequency concentration. The expression of the RSPWVD algorithm is as follows:

(4) $$\mathrm{RSPWV}D_{x}\left(t^{\prime },\omega^{\prime }\right)=\int_{-\infty }^{+\infty }\int_{-\infty }^{+\infty }\mathrm{SPWV}D_{x}\left(t,\omega \right)\times \delta \left(t^{\prime }-\hat{t}\left(t,\omega \right)\right)\delta \left(\omega^{\prime }-\hat{\omega }\left(t,\omega \right)\right)dtd\omega$$

where $\hat{t}\left(t,\omega \right)$ and $\hat{w}\left(t,\omega \right)$ represent the reassignment of energy centroids on the time–frequency plane. δ is the pulse function that concentrates the TF energy. In this paper, we adopt RSPWVD as the learning object of TFGM and select eight classic radar signals. Figure 1 shows the time–frequency images of 12 dual-component radar signals obtained using RSPWVD.

## 3. Methodology

Our dual-component radar signal recognition model, denoted by TFGM-RMNet, utilizes a TFGM module to replace the traditional TFT method to generate TF features in the time–frequency domain. It employs RMNet as the feature extraction module to extract local and global features from the TFR generated by TFGM. During the training phase of the model, we first preprocess the input noisy radar signal by normalization. Subsequently, the network weights learned by TFGM are used as basis functions to extract signal TF features, and the time–frequency domain features are reconstructed to generate clean TFR. In this process, we use RSPWVD to convert the time–frequency images of the clean dual-component radar signal as labels to facilitate the learning of the TFGM module. Finally, RMNet is used to extract deep features from the TFR. In this process, we use multiple loss functions to evaluate this multi-task learning model, thereby improving the model’s ability for time–frequency energy aggregation and classification performance. During the testing phase of the model, there is no need to use TFT tools, achieving a direct end-to-end process from input time-domain signals to output classification results.

### 3.1. Framework

**Figure 2:**
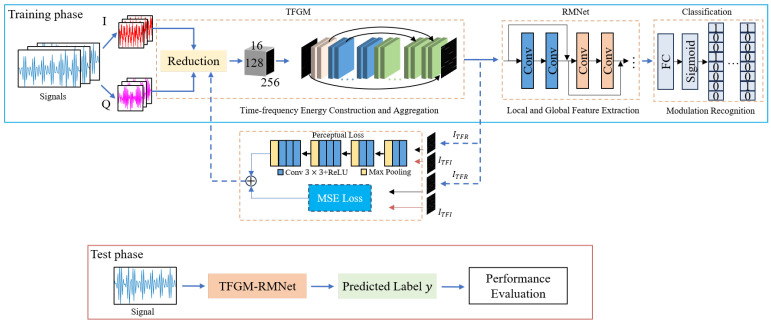
Proposed framework diagram. In the training phase, the dual-component modulated waveform is first decomposed into I/Q data, and the corresponding TFR is generated using TFGM. Then, the RMNet module extracts local and global features. Finally, the classifier is trained for multi-label classification. In the testing phase, when a modulated waveform is received, no multi-step operations are needed. By inputting the time-domain signal into TFGM-RMNet, the recognition result is directly output, achieving an end-to-end recognition process.

Our dual-component radar signal recognition framework is shown in Figure 2. It consists of a time–frequency feature generation module and a multi-label modulation recognition module.

The time-frequency feature generation module consists of a reduction module, an encoder, a decoder, and an upsampling layer. It extracts features from the original time domain signal and outputs the TFR of the signal. Using the TFGM module, there is no need to explicitly display and process the time-frequency image during the recognition process. The process from signal(noise)-TFR(clear) can be directly implemented, without the need to go through signal(noise)-TFI(noise)-TFI(denoise)-TFR(clear) as in existing work. The obtained TFR vector can be directly output to the next recognition module, building an end-to-end recognition process.

The multi-label modulation recognition module consists of RMNet and a multi-label classification layer. RMNet uses the locality and translation invariance of convolution to extract the underlying features of the input image, enhance the generalization ability of the overall model, and reduce the dependence of the subsequent attention module on the amount of data. The cascaded multi-head self-attention mechanism based on Transformer improves the quality of capturing global image feature context information. The multi-label classification layer maps the extracted features to each possible modulation category and outputs the predicted probability of each category. The predicted probability of each label can be mapped to the [0, 1] interval using the sigmoid function, indicating the probability of each category. By setting an appropriate threshold, the existence of each label can be judged, thereby achieving multi-label classification and the recognition of modulated signals.

### 3.2. Time–Frequency Representation Generation

**Figure 3:**
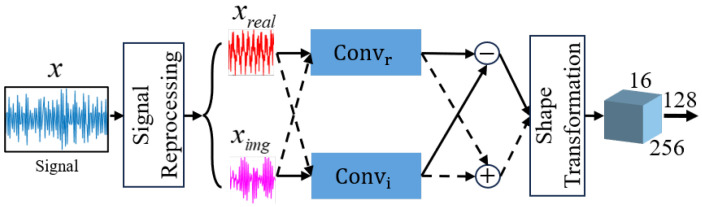
Reduction module structure diagram.

**Figure 4:**
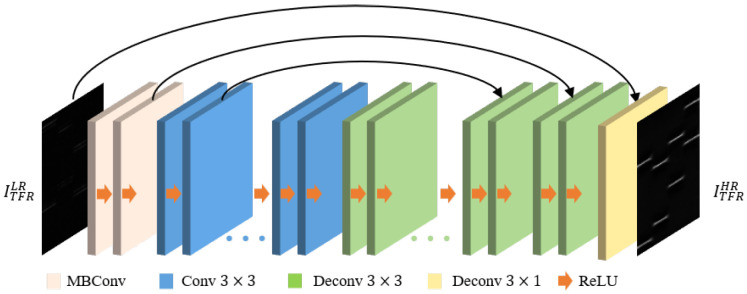
Encoder and decoder.

We constructed the TFGM module in the dual-component modulation recognition framework. TFGM learns TFT through reduction, encoder and decoder to obtain the ability to generate clean TFR. Figure 3 shows the reduction module, which mainly describes how the convolution operation is analogous to the short-time Fourier transform (STFT) to learn the Fourier basis to implement convolution to extract the frequency domain features of the time domain signal. Figure 4 illustrates the encoder and decoder, whose functions are to aggregate and reconstruct the time–frequency feature maps extracted by reduction [30].

#### 3.2.1. Reduction

The convolution operation in a neural network maps the local values within the receptive field to a point in the feature map, whereas in the Fourier transform, each frequency value *w* corresponds to the global time-domain information of the signal x(t) over the time axis from t=−∞ to t=∞, as shown in the following Equation (5). The Fourier transform can also be interpreted as a coordinate transformation in Hilbert space by selecting a set of orthogonal bases, thus achieving the conversion from the time domain to the frequency domain.

(5) $$F\left(w\right)=\int_{-\infty }^{+\infty }x\left(t\right)e^{-iwt}dt$$

(6) $$F\left(w\right)=\;<\vec{x},e^{iwt}>$$

where *t* is the time variable, *w* is the frequency variable, and F(w) is the frequency representation of x(t).

It can be seen that the convolution operation in neural networks and the Fourier transform share a certain similarity in their mapping approaches. To enable the Fourier transform to map only local signals, a window function is introduced to obtain the STFT:

(7) $$G\left(t,w\right)=\int_{-\infty }^{+\infty }g\left(u-t\right)x\left(u\right)e^{-jwu}du$$

where g(u−t) is the sliding window function.

The STFT analyzes the local time–frequency characteristics of a signal by sliding a window function over the time-domain signal, which is similar to the movement of the receptive field in neural networks. The convolution operation without a bias term is given by:

(8) $$Y_{m}\left(k\right)=\sum_{n=0}^{L_{c}-1}x_{m}\left(n\right)W\left(n,k\right)$$

where $L_{c}$ is the length of the convolution kernel, $x_{m}\left(\cdot \right)$ is the *m*th segment of the input signal, W(n,k) is the kernel weight. Based on the use of the window function g(u−t) in the STFT to extract a portion of the original signal x(u), the segmented signal $x_{m}\left(n\right)$ can also be written as g(n−m)x(n). Therefore, Equation (8) can also be described as:

(9) $$Y_{m}\left(k\right)=\sum_{n=0}^{L_{c}-1}g\left(n-m\right)x\left(n\right)W\left(n,k\right)$$

The above convolution formula is actually the general transformation TFR(m,k) of the discrete STFT with a window length of one-dimensional convolution kernel length $L_{c}$. From Equations (6), (7) and (9), we can see that the convolution operation updates the weight matrix W(n,k) to learn the basis in the transformation, thereby extracting TF features from the time-domain signal.

Initially, the input signals are preprocessed. The model takes 1 × 1024-dimensional complex signals as input and pads signals shorter than 1024 with zeros. Considering the large variation in the amplitude of different signals, normalization is applied to prevent the saturation of feature maps in early layers and accelerate convergence.

The reduction module maps the time domain signal to the TF feature. The input preprocessed signal $x_{n}\in^{n\times 1\times 1024}$ is split into I/Q data to form $x_{n}^{i,q}\in^{n\times 2\times 1024}$. We use the real part $Conv_{r}$ and imaginary part $Conv_{i}$ in the complex-valued one-dimensional convolution $H_{x}$ to perform moving convolution on the I/Q complex signal ($x=x_{\mathrm{real}}+x_{imag}$). According to Equation (8), we can obtain the operation of complex-valued convolution as follows:

(10) $$\begin{array}{c} \mathrm{IFR} \end{array}\begin{array}{c} =x_{n}H_{x}\\ =\left(x_{r}+jx_{i}\right)\left(Conv_{r}+jConv_{i}\right)\\ =\sum \left(x_{r}+jx_{i}\right)\left(W_{r}+jW_{i}\right)\\ =\left(\sum x_{r}W_{r}-\sum x_{i}W_{i}\right)+j\left(\sum x_{r}W_{i}+\sum x_{i}W_{r}\right) \end{array}$$

where, $W_{r}$ and $W_{i}$ are the real and imaginary parts. The convolution weight matrix W is continuously updated through the above complex-valued convolution process, guiding the network weights to adaptively learn the basis functions, and finally realizing the extraction of frequency features from the time domain signal.

#### 3.2.2. Encoder and Decoder

The encoder–decoder network integrates and aggregates TF features extracted by the reduction module to obtain aggregated TFR. The encoder, placed at the front of the network as a feature extractor, consists of two MBConv [31] blocks and several concatenated 2D convolutions. This structure aims to preserve the main components of TF features in the feature map while eliminating noise. The reduction module outputs feature maps $X\in R^{\left(C,H,W\right)}$. MBConv uses 1×1 convolutions with an expansion ratio of 1, as well as pointwise convolutions with kernel size 1×1 and depthwise convolutions with kernel size 3×3, to extract features from X and better capture and utilize its spatial and channel correlations. Each convolution layer in MBConv is followed by Leaky ReLU activation and batch normalization to introduce nonlinearity and enhance feature stability. The output feature $X\in R^{\left(2C,H/2,W/2\right)}$ then enters a series of concatenated convolutional layers with 3×3 kernels. Each convolution can be seen as further abstraction and refinement of the TF features from the previous layer, allowing the model to capture key features of different scales and complexities. ReLU activation follows each convolutional layer, transforming negative values to zero and preserving positive values to enhance the network’s nonlinear properties for learning complex data patterns and features.

Specifically, the deep feature extraction of the encoder consists of 2 MBConv layers and N(N=18) convolutional layers, and the feature extraction process can be expressed as:

(11) $$\begin{array}{c} F_{1}=\delta \left(\mathrm{MBConv}\left(X,W_{1},b_{1}\right)\right)\\ F_{2}=\delta \left(\mathrm{MBConv}\left(F_{1},W_{2},b_{2}\right)\right)\\ \vdots \\ F_{i}=\delta \left(W_{i}*F_{i-1}+b_{i}\right),\;i=3,4,\ldots ,20 \end{array}$$

where $W_{i}$ and $b_{i}$ represent the weight and bias parameters, and $F_{i}$ is the output feature map of the *i*-th layer of the encoder.

(12) $$\begin{array}{c} G_{1}=\delta \left(\mathrm{ConvTranspose}2d\left(G_{0},W_{1}^{T},b_{1}^{T}\right)\right)\\ G_{2}=\delta \left(\mathrm{ConvTranspose}2d\left(G_{1},W_{2}^{T},b_{2}^{T}\right)\right)+F_{18}\\ \vdots \\ G_{i}=\delta \left(\mathrm{ConvTranspose}2d\left(G_{i-1},W_{i}^{T},b_{i}^{T}\right)\right)+F_{20-i} \end{array}$$

(13) $${\mathrm{TFR}}_{\mathrm{out}}=\mathrm{ConvTranspose}2d\left(\delta \left(G_{20}+X\right)\right)$$

where $G_{0}$ is the input feature map of the decoder, which comes from the last layer output of the encoder. $G_{i}$ represents the output feature map of the *i*-th layer of the decoder, and every two deconvolutional layers receive a skip connection from the encoder. Finally, all the feature maps are aggregated and upsampled to the original spatial resolution using transpose convolutional layers, resulting in a TFR output size of 256×256×1, as shown in Figure 4.

### 3.3. Multi-Label Modulation Identification

RMNet adopts a structure that combines ResNet with cascaded multi-head self-attention, leveraging the strengths of both convolutional operations and self-attention mechanisms to achieve complementary advantages. Convolutional operations have limited receptive fields and translation invariance properties, which can lead convolutional networks to focus on local features while neglecting global context [32]. In contrast, the cascaded multi-head self-attention mechanism can connect information from any position, enabling the effective computation of long-range sequences and capturing dependencies between features across the entire global context. However, it has relatively poor modeling capabilities for 2D local data.

**Figure 5:**
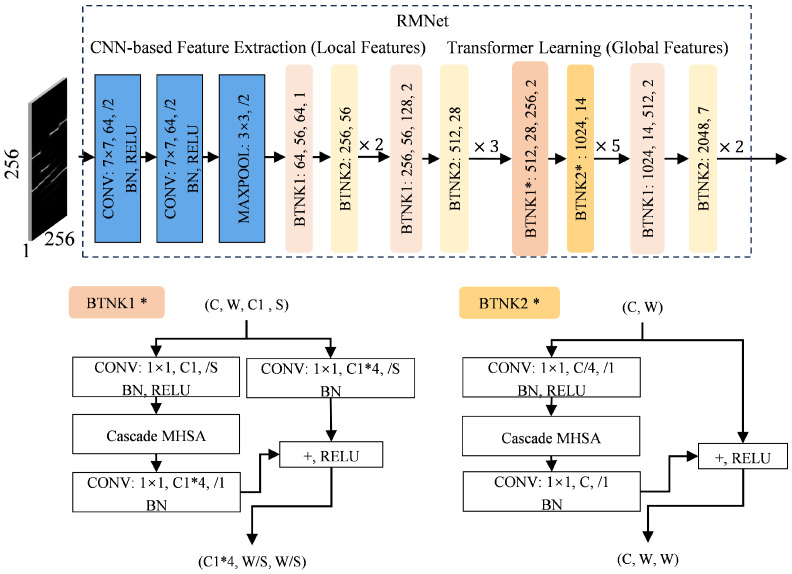
RMNet. The asterisks “*” in BTNK1* and BTNK2* in the image indicate layers with cascaded multi-head attention.

In the ResNet50 backbone network, layer1 and layer2 mainly extract low-level features, such as edges and textures. At this stage, local information in the feature maps is more critical, and traditional convolutional operations are better suited for extracting these features. The deeper layer4 mainly extracts high-level features, with a lower resolution of feature maps. Meanwhile, the mid-layer layer3 contains both local information and some global context information. Integrating cascaded multi-head self-attention modules into layer3 can effectively capture global dependencies and enhance the ability to model complex patterns. Specifically, we use six cascaded multi-head self-attention modules in layer3 to replace the traditional 3 × 3 spatial convolution. This design allows local features to be enhanced through convolutional operations while global dependencies are modeled through the self-attention mechanism, achieving a comprehensive analysis of features. The structure of RMNet is shown in Figure 5.

**Figure 6:**
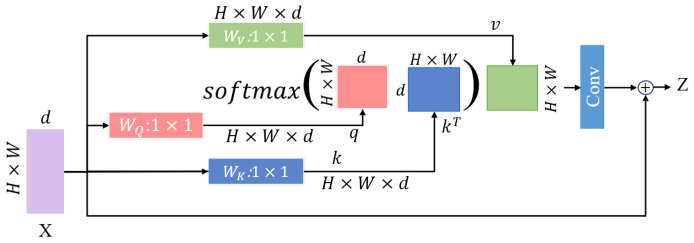
Cascaded multi-head attention.

Figure 6 shows the specific framework of the cascaded multi-head attention, where the input feature map $X\in R^{\left(H,W,d\right)}$, *H* and *W* are the height and width, respectively. Through learned linear transformations, the input X is mapped to query $Q=XW_{q}$, key $K=XW_{k}$, and value $V=XW_{v}$ [33]. The dot product of *Q* and *K* computes the attention score matrix $QK^{T}$. To stabilize gradients, a scaling factor $\frac{1}{\sqrt{d_{k}}}$ is introduced, where $d_{k}$ is the dimension of each head. This scaling factor helps prevent excessively large scores, making the gradient of the Softmax function more stable. The scaled dot product matrix is then input to the Softmax function to calculate the attention activation map A. Finally, by multiplying the attention activation map A with the values *V*, the final output Z is obtained. The specific process is as follows:

(14) $$\begin{array}{c} Z_{i}=\mathrm{Attention}\left(Q_{i},K_{i},V_{i}\right)\\ =\mathrm{softmax}\left(\frac{Q_{i}{K_{i}}^{T}}{\sqrt{d_{k}}}\right)V_{i} \end{array}$$

(15) $$Z=\mathrm{Concat}\left(Z_{1},Z_{2},\ldots ,Z_{h}\right)W_{O}$$

To further integrate the attention output features, a convolutional layer is added to perform feature transformation while preserving the spatial structure. Then, a skip connection adds the input feature map X to the output after convolution and Dropout, helping the model retain shallow features while learning new ones, merging new and old features, enhancing the model’s representational capability, and mitigating the vanishing gradient problem. Finally, layer normalization is applied after the skip connection to avoid excessive differences in feature distributions between different sub-layers and further stabilizing gradient flow. The specific process is shown below:

(16) $$Z^{\prime }=Conv\left(Z\right),$$

(17) $$Z^{\prime \prime }=D\left(Z^{\prime }\right)+X,$$

(18) $$Z^{\prime \prime \prime }=LN\left(Z^{\prime \prime }\right),$$

where D(·) is the dropout operation and LN(·) refers to the layer normalization operation.

### 3.4. Training

Our recognition framework is a multi-task learning model that trains corresponding tasks using different loss functions. To enable the TFGM module to generate high-quality TFRs, the choice of a loss function is crucial for achieving a good generation performance. When the TFGM learns to generate TFRs from time-domain waveforms, it transforms a one-dimensional signal matrix into a two-dimensional image matrix. In this process, the TF features only occupy a small portion of the generated two-dimensional TFR matrix, making the effective feature regions sparse within the entire TFR. Therefore, a pixel-level reconstruction loss is necessary to address this issue. The pixel-wise MSE loss is calculated as follows [34]:

(19) $$L_{MSE}=\frac{1}{H*W}\sum_{i=1}^{H}\sum_{j=1}^{W}{\left(I_{ij}-{\hat{I}}_{ij}\right)}^{2}$$

where H∗W is the total number of pixels, and $I_{ij}$ and ${\hat{I}}_{ij}$ represent the gray values of the (i,j)th pixel in the label time–frequency image and the generated time-frequency image, respectively.

However, the MSE loss function only focuses on pixel-level differences and is insufficient for capturing the details and linear structures of the target signal in the TFR. The shape and contour information of the signal in the TFR are critical features for the subsequent recognition network. To make the generated TFR closer to the ideal TFI, we introduce perceptual loss that measures the similarity between the generated image and the target image by comparing their feature representations in the intermediate layers of a pre-trained neural network. We employ the pre-trained VGG16 network for feature extraction. For the *j*th layer of the VGG16 network, the image loss can be expressed as:

(20) $$L_{\mathrm{feat}}^{\varphi ,j}=\frac{1}{C_{j}H_{j}W_{j}}{∥\varphi_{j}\left(I\right)-\varphi_{j}\left(\hat{I}\right)∥}_{2}^{2}$$

where φ represents the VGG16 network, *j* is the layer index, $\varphi_{j}\left(\hat{I}\right)$ and $\varphi_{j}\left(I\right)$ are the features of the generated and target images passed through the *j*th layer of the VGG16 network, and $C_{j}$, $H_{j}$, $W_{j}$ represent the channels, height, and width of the features of the *j*th layer, respectively.

The overall perceptual loss is obtained by summing the losses of all layers, represented as [35]:

(21) $$L_{fea}=\sum_{j=0}^{N}L_{\mathrm{feat}}^{\varphi ,j}\left(I,\hat{I}\right)$$

where *N* is the total number of layers in the VGG16 network.

For the multi-label modulation recognition task, we use binary cross-entropy loss with logits as the loss function to compute the loss between the predicted labels and the given labels. It combines the Sigmoid layer and binary cross-entropy loss into one component, which is numerically more stable compared to using simple Sigmoid and binary cross-entropy loss separately. The loss function is described as follows:

(22) $$L_{CE}=-\frac{1}{N}\sum_{i=1}^{N}\sum_{c=1}^{C}\left[w_{c}y_{ic}\log\left(\delta \left(p_{ic}\right)\right)+\left(1-y_{ic}\right)\log\left(1-\delta \left(p_{ic}\right)\right)\right]$$

(23) $$\delta \left(p_{ic}\right)=\frac{1}{1+e^{-p_{ic}}}\;\;\;\;\;\;\left(c\in 1,\ldots ,8\right)$$

where $w_{c}$ is the weight of class *c*, $y_{ic}$ is the true label of the sample, $p_{ic}$ is the logits output by the model, and $\delta (p_{ic})$ is the sample probability output.

**Figure 7:**
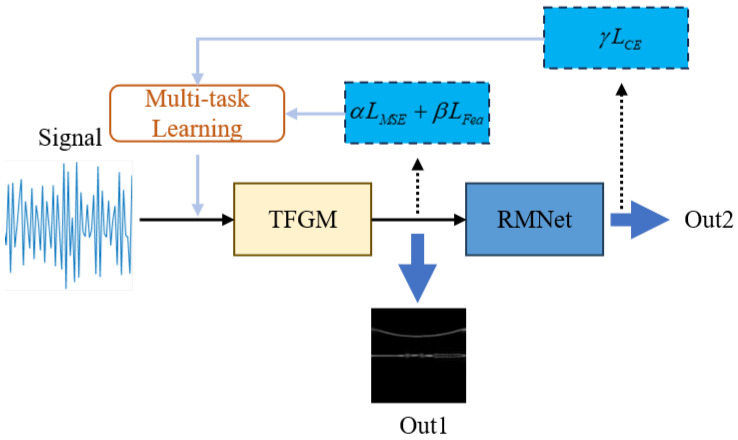
Training mechanism of network.

Figure 7 illustrates the training process of TFGM-RMNet. The TFGM-RMNet recognition framework integrates TFR generation tasks and modulation recognition tasks. On one hand, the input time-domain signal passes through the TFGM module, calculating $L_{MSE}$ and $L_{Fea}$ using Equations (19) and (21), respectively, to obtain TFR. On the other hand, the TFR output by the TFGM module is processed by the RMNet feature extraction network to obtain the corresponding feature values $x_{c}$. The feature values are then used in Equation (22) to calculate $L_{CE}$, resulting in the final classification result Out2. During the testing process, only forward propagation is executed to output classification results and the expected TFR.

## 4. Experiments

For the experiments, in order to verify the effectiveness and reliability of the proposed TFGM-RMNet network, we provide a series of experiments. Section 4.1 introduces the dataset design in detail. Subsequently, Section 4.2 gives the evaluation metrics. Section 4.3 shows the generalization under different SNR conditions. Next, Section 4.4 evaluates the quality of TFR generated by TFGM. Finally, Section 4.5 gives the accuracy comparison of the proposed model with existing advanced models.

### 4.1. Dataset

To fully consider the practical scenarios of radar in electronic warfare, eight standard waveforms were studied, including BPSK, 4FSK, LFM, FRANK, 2FSK, SFM, EQFM, NS. The eight modulation formats and their corresponding parameters are shown in Table 1, where the sampling frequency $f_{s}$ and the number of samples *N* are set to 200 MHz and 1024, respectively.

The radar signal is modeled and the dataset is constructed according to the signal parameters in Table 1. Both the training set and the test set consist of two-component modulated signals, which are simulated by two randomly selected modulated signals, with a total of 36 combinations. The SNR of the training set ranges from −12 dB to 10 dB, and 30 samples are generated for each two-component modulation type at different SNR. A total of 1080 overlapping two-component signal samples are generated at each SNR with an interval of 2 dB, and a total of 12,960 samples in the training set. Similarly, for the test set, 360 samples are generated at the same SNR level, and the signals are randomly overlapped. When testing the recognition accuracy at each SNR, the proportions of training, test, and validation data are, respectively, 97.7%, 2.8%, and 2.8%. The learning object in TFGM is obtained by performing traditional RSPWVD TFT on noise-free signals and reset 256×256.

### 4.2. Evaluation Metrics

For multi-label evaluation, we use a variety of methods to evaluate and understand the performance of the TFGM-RMNet network, including accuracy, precision, recall, and F1 score.

To measure the model’s predictive power for each label, we evaluated the accuracy (ACC) and F1-score for each label separately. The ACC of each label is defined as the ratio of correctly predicted samples to the total number of samples:

(24) $$\mathrm{ACC}=\frac{1}{n}\sum_{i=0}^{n}\left(y_{i}^{j}={\hat{y}}_{i}^{j}\right)$$

where *n* is the total number of samples, $y_{i}$ is the set of true labels for the *i*-th sample, ${\hat{y}}_{i}$ is the set of predicted labels for the *i*-th sample, and *j* represents the specific label.

Precision is defined as the ratio of positive predictions that are truly positive, i.e., TP/(TP + FP), where TP is a true positive and FP is a false positive. It indicates how many of all samples predicted by the model as positive are actually positive:

(25) $$\mathrm{Precision}=\frac{\sum_{i=1}^{n}\left|y_{i}^{j}\cup {\hat{y}}_{i}^{j}\right|}{\sum_{i=1}^{n}{\hat{y}}_{i}^{j}}$$

Recall is defined as the ratio of positive samples that are correctly predicted as positive, i.e., TP/(TP + FN), where FN is a false negative. It indicates how many of the samples that are actually positive are correctly predicted as positive by the model:

(26) $$\mathrm{Recall}=\frac{\sum_{i=1}^{n}\left|y_{i}^{j}\cup {\hat{y}}_{i}^{j}\right|}{\sum_{i=1}^{n}y_{i}^{j}}$$

F1-*score* is a combination of precision and recall, and is an indicator used to balance the model’s precision and recall. The higher the F1-*score*, the better the model performs in balancing precision and recall:

(27) $$F1-score=\frac{2\times \mathrm{Precision}\times \mathrm{Recall}}{\mathrm{Precision}+\mathrm{Recall}}$$

### 4.3. Generalization Under Different SNR Conditions

**Figure 8:**
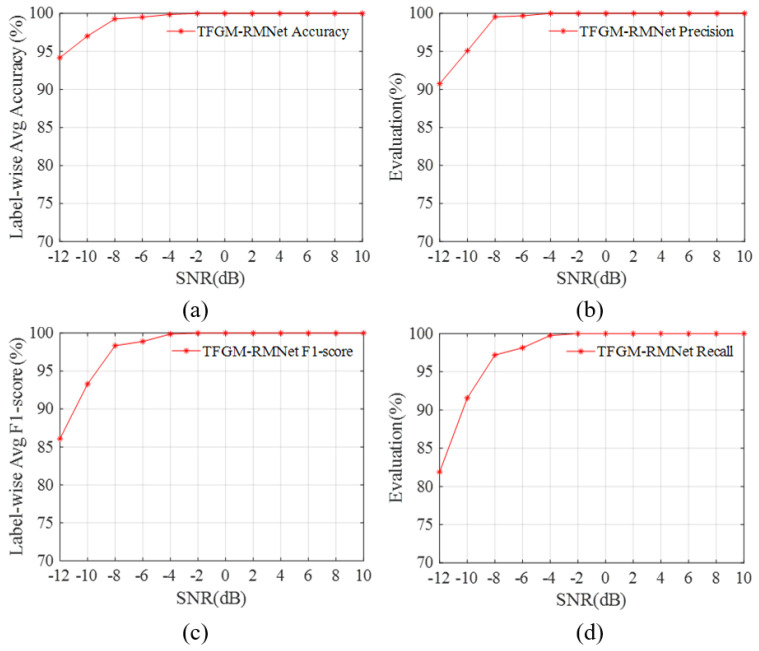
Performance of TFGM-RMNet over the SNR range of —12 to 10 dB. (**a**) Labelwise average accuracy. (**b**) Precision. (**c**) Labelwise average F1-score. (**d**) Recall.

As a critical parameter in signal processing and communication systems, SNR directly affects signal quality and recognition performance. In radar signal recognition tasks, signals in low SNR environments are usually accompanied by high noise levels, making it more challenging to accurately extract signal features. We study the performance of various metrics in the SNR range of −12–10 dB. Figure 8 shows the overall average accuracy, precision, and recall of the proposed method for labels, respectively, and also uses the F1-score to measure the combined results of precision and recall.

From Figure 8, it can be seen that the four indicator parameters first increase rapidly with the increase in SNR and then tend to stabilize. The proposed framework performs well in the tested SNR range, and has good performance even under low SNR conditions. When the SNR is −10 dB, the overall average accuracy, precision, and recall all exceed 90%, with the recognition accuracy reaching 97%. Even when the SNR is −12 dB, the average recognition accuracy is still above 94%, demonstrating that TFGM-RMNet has a good recognition effect for dual-component radar signals. The dual-component test set usually faces the problem of label imbalance. We use the F1-score to measure the combined results of precision and recall. As shown in Figure 8c, the F1 score is close to 99% at −6 dB and reaches 100% at −4 dB. Even at an SNR of −10 dB, it can still achieve more than 93%. This indicates that the proposed network can handle the problem of label imbalance well under low SNR conditions.

**Figure 9:**
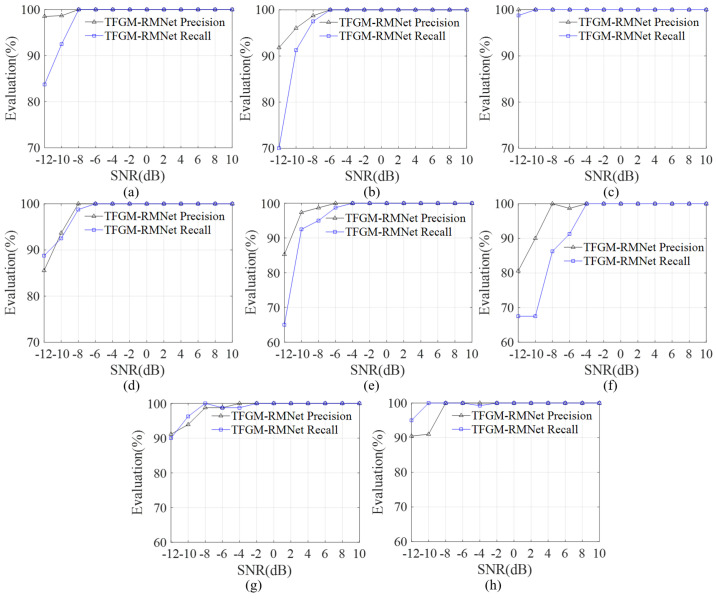
Labelwise performance of the TFGM-RMNet framework for different modulation schemes. (**a**) 4FSK. (**b**) BPSK. (**c**) LFM. (**d**) FRANK. (**e**) EQFM. (**f**) SFM. (**g**) NS. (**h**) 2FSK.

Figure 9a–h presents the label accuracy and recall rate of our proposed framework under different SNR conditions. When SNR > −8 dB, only the recall rate of the SFM signal is above 90% among the eight types of signals, while the recall rates of the remaining seven types of signals are all above 98%, and the label accuracy is close to 100%. When the SNR is −4 dB, the label accuracy and recall rate for all eight types of signals are close to 100%, proving that the proposed framework achieves overall satisfactory performance under higher SNR conditions. Under low SNR conditions, the label accuracy of FRANK, EQFM, and SFM is relatively low. By examining the TFI labels, we can see that the SFM signal has insufficient energy and indistinct signal contours on the time–frequency image, resulting in TFGM’s inability to generate high-quality TFRs, which leads to poor reconstruction performance and subsequently affects the recognition performance at low SNR. However, when the SNR is −12 dB, the precision values for these three signals are 85.54%, 85.25%, and 80.60%, respectively. However, when the SNR is above −10 dB, these signals achieve precision rates of over 95%. In addition, the recall rates of LFM and 2FSK are relatively high compared to other signals. As shown in Figure 9c,h, when the SNR is −12 dB, the recall rate reaches above 95%. We found that, under extremely low SNR conditions, TFGM often struggles to generate satisfactory TFRs for signals severely affected by noise and with weak time–frequency clustering characteristics. Experiments show that further research on some pre-processing algorithms for time–frequency analysis or specialized kernel designs can help obtain ideal time–frequency images and use clearer features as labels, thereby fitting and generating higher-quality time–frequency images.

### 4.4. Time–Frequency Image Generation and Denoising Performance

**Figure 10:**
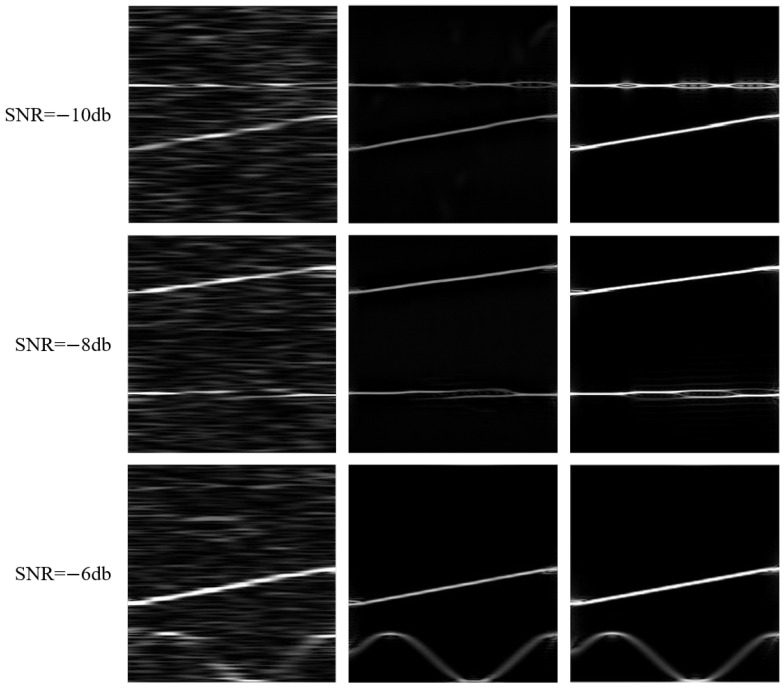
The first, second, and third columns are, respectively, the RSPWVD time–frequency image, the time-frequency image generated by TFGM, and the ideal time–frequency image under three SNRs.

In order to evaluate the effectiveness of the TFGM module in generating time–frequency images for the original signal, Table 2 and Table 3 show the structural similarity index measurement (SSIM) and peak signal-to-noise ratio (PSNR) of the TFI generated by RSPWVD and the TFI generated by TFGM at −10 dB, −8 dB, and −6 dB SNRs. It can be seen from the table that as the SNR increases, the SSIM and PSNR values of the TFI generated by RSPWVD and the TFI generated by TFGM also gradually increase. When the SNR is −10 dB, the same time-domain signal is reconstructed by the TFGM model to generate the TFR, resulting in a structural similarity that is 4 times higher than that of the TFI generated by RSPWVD. When the SNR is −8 dB, the SSIM and PSNR values of the TFI generated by TFGM are very close to those at −10 dB. When the SNR is −6 dB, the SFM signal is significantly affected by noise, causing the signal features to be blurred. Therefore, the metrics for the TFI generated by RSPWVD are lower, decreasing compared to −8 dB. However, the TFGM module demonstrates satisfactory generalization performance, with SSIM and PSNR reaching 0.9 and 24, respectively. Since the SFM signal is greatly affected by noise, the index values are relatively low. Examples of TFI generated by RSPWVD and TFI generated by TFGM are shown in Figure 10. SNR = —10 dB, the TFI generated by TFGM has some feature loss, and some phase features of the BPSK signal are not generated, but satisfactory generation and denoising effects are still obtained.

**Figure 11:**
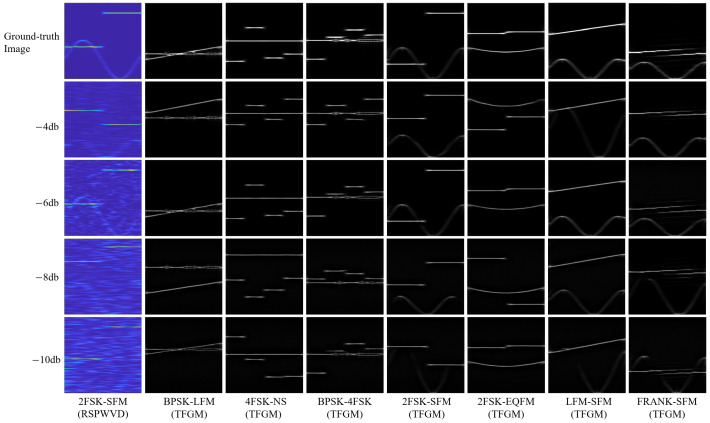
TFIs generated by RSPWVD and TFGM at different SNRs.

TFGM can simulate traditional TFT by generating clean TFR from noisy signals. As shown in Figure 10, the TFR generated by TFGM is comparable in imaging style to the ideal TFI based on RSPWVD. Figure 11 shows the TFI images drawn using the dual-component radar signal TFR generated by TFGM at different SNRs. The first row is the ground-truth image when the SNR is −6 dB, the leftmost column is the 2FSK-SFM TFI randomly generated by the traditional time–frequency transform RSPWVD, and the rest are TFI generated by TFGM. TFGM acts directly on the signal and can fully utilize information in the signal domain to optimize the existing TFT process as much as possible. Even at extremely low SNR, TFGM can still generate clean TFR, and as SNR improves, the features of the generated TFR become increasingly clear.

### 4.5. Recognition Accuracy Comparison

To further evaluate the performance of TFGM-RMNet, we conduct ablation experiments to obtain the recognition performance of TFGM-RMNet and RMNet, and compare their recognition performance with related existing works. For a fair comparison, we fine-tune the existing models and test them on the same dual-component training and test sets as RMNet. These methods are as follows:

(1) DCNN-MLL: MLL-DCNN [22] was proposed for dual-component recognition based on the CNN model MBConv, and uses a fully connected layer with thresholds for multi-label classification.
(2) DCNN-MIML: DCNN-MIML [21] was proposed for the recognition of overlapping radar waveforms using TFI and employs a CNN-based VGG16 MIML classifier for multi-component modulation recognition.
(3) DCNN-RAMIML: DCNN-RAMIML [36] adopts the CNN model ResNet34 and uses channel and spatial RA to improve the MIML classifier.

**Figure 12:**
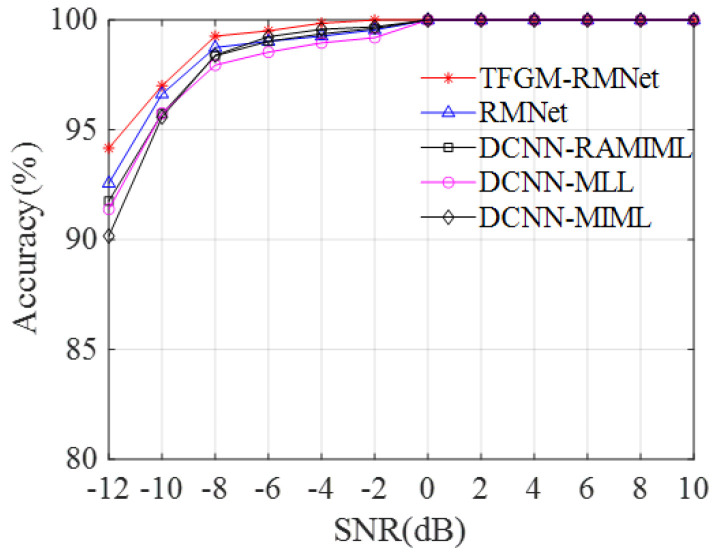
The recognition accuracy of various methods varies with the SNR.

Figure 12 depicts the variation in the recognition accuracies of TFGM-RMNet, RMNet, DCNN-RAMIML, DCNN-MLL, and DCNN-MIML with the increase in SNR. As shown in the figure, the recognition performance of the five methods increases rapidly with increasing SNR and then stabilizes. Notably, the recognition accuracy of TFGM-RMNet increases significantly faster than the others, especially at lower SNRs. When the SNR is −12 dB, TFGM-RMNet is 2.4%, 2.78%, and 4% higher than DCNN-RAMIML, DCNN-MLL, and DCNN-MIML, respectively, and 1.6% higher than RMNet. This is because, at low SNRs, the signals are greatly affected by noise, and the TFI generated by traditional TFA is also disturbed by noise, reducing the effective features in the TFI and leading to lower average recognition accuracy. In contrast, the basis function of TFGM adaptive learning can obtain more effective frequency domain information, thereby generating high-quality TFRs, making the subsequent feature extraction process more accurate. Additionally, the RMNet module, which combines the convolution operations and multi-head self-attention mechanisms, can capture both local and global features simultaneously, enhancing the model’s recognition capability in complex signal environments. When the SNR is −4 dB, TFGM-RMNet achieves nearly 100% accuracy. At an SNR of 0 dB, other methods also reach 100%. For SNRs that are less than 0 dB, the TFGM-RMNet model demonstrates better recognition performance.

**Figure 13:**
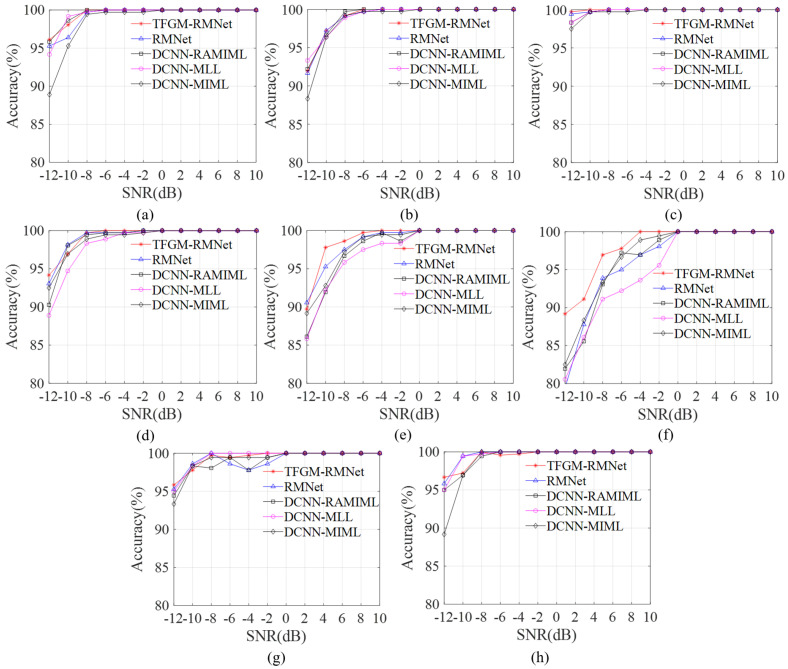
Recognition accuracy of each modulation scheme at different SNRs. (**a**) 4FSK. (**b**) BPSK. (**c**) LFM. (**d**) FRANK. (**e**) EQFM. (**f**) SFM. (**g**) NS. (**h**) 2FSK.

Figure 13 shows the recognition accuracy of eight signals of five algorithms at different SNRs. As can be seen from the figure, the average recognition performance of the five methods improves with increasing SNR and gradually stabilizes at higher SNR levels. At higher SNRs, the accuracy of the five algorithms is similar. However, at lower SNRs, there are significant differences in recognition accuracy. Notably, the proposed TFGM-RMNet achieves a recognition accuracy of over 89% for various signals, and its recognition accuracy for the eight signals is close to 100% when the SNR is greater than −6 dB. Due to the distinct intra-pulse characteristics of LFM signals, when the SNR is −12 dB, the five algorithms TFGM-RMNet, RMNet, DCNN-RAMIML, DCNN-MLL, and DCNN-MIML have the best recognition accuracy, which are 99.72%, 99.44%, 98.33%, 98.33% and 97.50%, respectively; for SFM signals, the accuracies are 89.17%, 79.44%, 81.94%, 80.56% and 82.50%, respectively, and TFGM-RMNet is 9.73%, 7.23%, 8.61% and 6.67% higher than the other four methods. This is because TFGM can obtain better TFR. As shown in Figure 11, under low SNR, SFM is greatly affected by noise, resulting in the signal features in the time–frequency image being almost submerged by noise. TFGM can generate clean TFR from noisy SFM time domain signals and reduce noise interference. In addition, RMNet has a better recognition accuracy for LFM, FRANK, EQFM, and 2FSK signals than the other three algorithms. Therefore, compared with other methods, the method proposed in this paper shows a better recognition performance, especially under low SNR.

## 5. Discussion

The proposed TFGM-RMNet model reformulates the collaborative scheme of TFT and CNN for dual-component radar signal recognition by replacing the traditional TFA with the deep TFA module TFGM to improve the recognition accuracy. Experimental results show that, compared with existing methods, our model has a better recognition performance under low SNR, and the TFGM module also exhibits good TFR generation and denoising capabilities. However, there are still some limitations of our model. First, compared with the traditional TFA, the TFGM module can obtain cleaner TFR under low SNR, but this is achieved at a high computational complexity. Second, although the convolution in the TFGM module can learn multiple basis functions, the fixed-size convolution kernel used will affect the learning of radar time series components of different durations, thereby limiting the diversity and effectiveness of the basis functions learned by the weight matrix. Based on the idea that the window size decreases with the increase in frequency in LSWA and CWT, in the future, we will design a dynamically adaptive variable-size convolution kernel so that the convolution kernel weights can learn more diverse and complete basis functions to better adapt to the learning of radar time series components of different durations. In addition, we also plan to study model compression to further reduce computational complexity.

## 6. Conclusions

In this paper, we propose a dual-component modulation recognition framework, named TFGM-RMNet, which combines the TFR generation task with the dual-component modulation recognition task and achieves excellent results. In the proposed framework, with the focus on signal domain information, TFGM uses the weight matrix of the convolution in the Reduction module to learn the basis function to extract the TF features. It finally generates the TFR by aggregation and reconstruction by encoder and decoder to generate TFR. RMNet combines ResNet with a cascaded multi-head attention mechanism to effectively extract both local and global features from the TFR, ultimately producing category predictions. During the testing phase, the end-to-end TFGM-RMNet network directly generates the final recognition results from the received signals without requiring the separate steps of TFT, denoising, feature extraction, and classification as in the existing methods. This end-to-end approach reduces the risk of introducing errors and optimizes traditional multi-class radar signal recognition methods. Due to TFGM’s ability in generating high-quality TFRs under low SNR conditions and RMNet’s excellent capacity in capturing global and local features, TFGM-RMNet outperforms the existing methods DCNN-RAMIML, DCNN-MLL and DCNN-MIMLand achieves good recognition performance even under low SNR, as demonstrated in the experiment results. In the future, our work will extend the two-component modulation recognition to multi-component radar signals in high-density electromagnetic environments.

## Figures and Tables

**Table 1:** Simulation radar signal parameters.

Signal Type	Parameter	Range
ALL	Sampling frequency	1 ($f_{s}=200$ MHz)
4FSK	Carrier frequency $f_{1}$ to $f_{4}$	U(0.1,0.4)
BPSK	Carrier frequency $f_{c}$	U(0.1,0.4)
	Barker codes $N_{c}$	{7,11,13}
LFM	Carrier frequency $f_{c}$	U(1/4,1/2)
	Bandwith *B*	U(1/16,1/8)
FRANK	Carrler frequency $f_{c}$	U(1/8,1/4)
	Phase number *M*	[4,7]
2FSK	Carrier frequency $f_{1}$ to $f_{2}$	U(0.01,0.46)
NS	Carrler frequency $f_{c}$	U(0.1,0.4)
EQFM	Carrler frequency $f_{c}$	U(0.1,0.4)
	Bandwith *B*	U(0.1,0.3)
SFM	Carrler frequency $f_{c}$	U(0.001,0.0015)
	Bandwith *B*	U(0.05,0.25)

**Table 2:** Comparison of SSIM between RSPWVD TFI and TFI generated by TFGM.

SNR	−10 dB	−8 dB	−6 dB
RSPWVD TFI	0.1151	0.1246	0.0745
TFGM TFI	0.4986	0.5044	0.904

**Table 3:** Comparison of PSNR between RSPWVD TFI and TFI generated by TFGM.

SNR	−10 dB	−8 dB	−6 dB
RSPWVD TFI	17.91	18.52	17.73
TFGM TFI	19.49	19.54	24.07

## Data Availability

The data are available from the corresponding author upon reasonable request.
